# A new detection method for a newly revealed mechanism of pyrethroid resistance development in *Varroa destructor*

**DOI:** 10.1007/s00436-015-4627-4

**Published:** 2015-07-26

**Authors:** Aneta Strachecka, Grzegorz Borsuk, Krzysztof Olszewski, Jerzy Paleolog

**Affiliations:** Department of Biological Basis of Animal Production, Faculty of Biology and Animal Breeding, University of Life Sciences in Lublin, Akademicka 13, 20-950 Lublin, Poland

**Keywords:** *Varroa destructor*, PCR-SSCP, Acaricides, Mitochondrial cytochrome oxidase I (COI), Drug resistance

## Abstract

The *Varroa destructor* mite has recently displayed an ever increasing resistance to new drugs, contributing to CCD proliferation. This work was aimed at determining new viable methods for identifying the pyrethroid resistance of *V. destructor* and DNA methylation in resistant and sensitive mites. DNA was extracted from *Varroa* mites. Nucleotide changes in the DNA of pyrethroid-resistant, pyrethroid-sensitive, and control mites were identified with polymerase chain reaction single-strand conformation polymorphism (PCR-SSCP) in the case of five mitochondrial gene fragments. More bands were observed in the drug-resistant mites than in the other two groups. Sequencing confirmed these observations. Decreased global DNA methylation levels were observed in the pyrethroid-resistant mites. There exists a previously undescribed mechanism of pyrethroid resistance development in *Varroa* mites. The PCR-SSCP methods can be considered and further developed as useful tools for detecting *V. destructor* resistance.

## Introduction

**Fig. 1:**
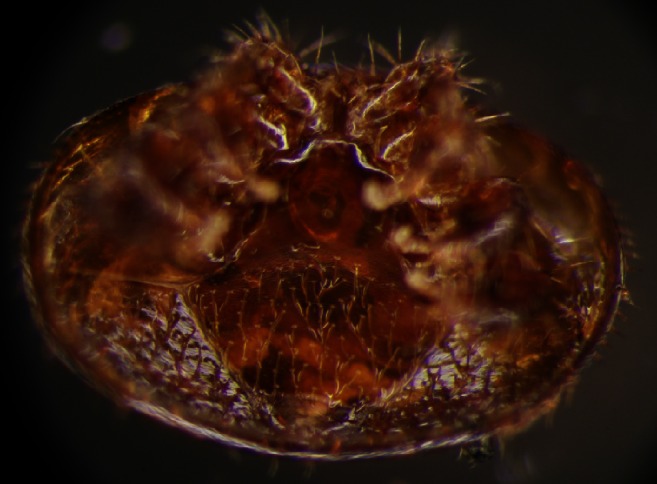
Venteral view of a *Varroa destructor* mite

*Varroa destructor* parasite (Fig. 1) is the crucial, worldwide problem of apiculture. It is believed to be one of the main reasons for honeybee colony depopulation (Rosenkranz et al. 2010). The parasites not only weaken and damage their hosts but also transfer viruses that lead to morphological malformations and suppress host vigor, life span, flight abilities, and forager orientation (Schneider and Drescher 1987; Koch and Ritter 1991; Romero-Vera and Otero-Colina 2002; Garedew et al. 2004; Kralj and Fuchs 2006; Maggi et al. 2009; Borsuk et al. 2012). Therefore, many acaricides are in use to control the infestation of honeybees by *V. destructor*. Despite high initial acaricide performance, the adaptive potential of *V. destructor* eventually leads to the appearance of the first acaricide-resistant individuals that are capable of reproduction. It may occur even after a few years from the beginning of treatment (Mathieu and Faucon 2000; Maggi et al. 2010, 2012). Watkins (1997) found that the higher the number of *Varroa* generations raised under tau-fluvalinate influence, the greater percentage of the *Varroa* population becomes more resistant to this synthetic pyrethroid used around the world to control honeybee infestation by *Varroa* parasites.

The resistance within the parasite populations has a multifactorial character. First, it is associated with increased tau-fluvalinate detoxification abilities mediated by cytochrome P-450 monooxygenases in some parasites. Martin (2004) described the *Varroa* cuticle resistance as the second resistance mechanism. Our former studies confirmed high activities of proteases on cuticles of the tau-fluvalinate-resistant *Varroa* mites in comparison to the tau-fluvalinate-sensitive ones (Strachecka et al. 2013). Wang et al. (2002) and Liu et al. (2006) described the third resistance mechanism: Tau-fluvalinate causes point mutations in mitochondrial DNA (mtDNA) and thus blocks sodium channels, which increases *Varroa* tolerance of this pyrethroid. Yukioka et al. (1998) and Van Leeuwen et al. (2008) also discovered that *Varroa* resistance to this acaricide is associated with mutations of mitochondrial genes. Such mutations result in an exchange of amino acids (e.g., leucine-phenylalanine) which leads to a change in the folding of proteins connected with the functioning of the sodium channel (Miyazaki et al. 1996; Williamson et al. 1996; Dong 1997; Guerrero et al. 1997; Lee et al. 1999; Park et al. 1997; Wang et al. 2002, 2003). These mtDNA mutations easily spread in the *Varroa* population because of the kin reproduction strategy of the mites (brothers mate with their sisters; Cornuet et al. 2006).

Mite resistance to acaricides, including tau-fluvalinate resistance, was mainly determined with the method of Milani (1995). It is difficult, however, to compare tau-fluvalinate toxicity evaluated by the Milani and the other methods (Hillesheim et al. 1996; Johnson et al. 2010) since, in these methods, the mortality and resistance of mites were assessed by applying different mediums, humidities, and temperatures.

Taking into account all the findings on *V. destructor* resistance against acaricide treatment, we believe that there is an urgent need (1) to develop new methods of resistance detection (including potential resistance) based on the assessment of parasite mtDNA mutation changes and (2) to expand the study of *Varroa* resistance mechanisms against acaricides, taking into consideration epigenetic mechanisms, since mtDNA mutations do not fully explain the parasite response/plasticity during acaricide treatment. Ultimately, the fast but variable and environment-dependent development of *Varroa* pyrethroid resistance can find a new explanation (Holliday and Grigg 1993; Xia et al. 2012; Flores et al. 2013). The aim of this study was (1) to develop a new method for detecting pyrethroid resistance of *V. destructor* based on polymerase chain reaction single-strand conformation polymorphism (PCR-SSCP) involving five mitochondrial genes. PCR-SSCP is a simple and effective technique detecting changes in the nucleotide sequence of PCR products (2) to reveal the existence of a yet undescribed mechanism of *V. destructor* pyrethroid resistance connected with nuclear DNA methylation.

## Material and method

One hundred *V. destructor* females were sampled from drone brood combs acquired from eastern Poland apiaries at which acaricides containing tau-fluvalinate (150 mg tau-fluvalinate per colony) had been applied. The females were divided into two unequal parts containing 75 and 25 of them, respectively. In the first part, the 75 females were divided into tau-fluvalinate-sensitive and tau-fluvalinate-resistant using test media soaked with tau-fluvalinate according to the Milani (1995) method. In the second part, 25 females were tested on pure, media that were not soaked with tau-fluvalinate, thus representing the control. Subsequently, the mites (samples) belonging to the resistant, sensitive, and control parasite groups were marked with special codes (blind samples) and refrigerated in germ-free bags at −24 °C. After defrosting, mite DNA was extracted using the DNeasy Blood & Tissue Kit (Qiagen) according to the producer’s procedure. Extracted DNA samples were stored at −25 °C. DNA quantification was performed spectrophotometrically by measuring the absorbance at 230, 260, and 280 nm with a BioPhotometer (Eppendorf). The DNA samples were used both for the PCR and the global DNA methylation analyses.

### The polymerase chain reaction

Five fragments from the following mitochondrial genes: cytochrome oxidase I, II, and III, ATP synthase 6, and cytochrome b (COI 320 bp, COI 929 bp, COII-ATP6 775 bp, ATP6-COIII 818 bp, and CytB 958 bp) were amplified in the PCR using the Qiagen Taq PCR Core Kit. The PCR primers (Table 1) were developed from the sequence of the complete mtDNA genome of *V. destructor* (GenBank Accession No. AJ493124.1; Evans and Lopez 2002; Navajas et al. 2002). The reaction mixture for a single sample contained 6 μl DNA, 3.5 μl 10× PCR buffer, 7 μl Q buffer, 5.33 μl MgCl_2_, 0.58 μl of each dNTP, 0.33 μl primers 1 and 2, and 1 U polymerase. The eventual volume of the sample was 30 μl. The amplification of the PCR products was carried out onboard Labcycler SensoQuest Biomedical Electronics (Syngen) thermocycler in accordance with the following thermal-temporal profile: preliminary denaturation at 94 °C for 3 min; subsequently, a program of 36 repetitive cycles was employed—denaturation at 94 *°*C for 1 min, attachment of the primers at 45 °C for 1 min, and annealing of the primers at 72 °C for 1 min. The final annealing of the primers was conducted at 72 °C for 10 min. The PCR products were divided into two sets: 1 for SSCP and 2 for the sequencer.

**Table 1:** Amplified DNA fragments for the detection of tau-fluvalinate-sensitive and tau-fluvalinate-resistant *V. destructor*

Gene	Primer name	Primer sequence (5′-3′)	Fragment size (bp)	Literature
COI	V51	GTAATTTGTATCAAAGAGGG	320	Warrit et al. 2006
	V1400	CAATATCAATAGAAGAATTAGC		
COI	10KbCOIF1	CTTGTAATCATAAGGATATTGGAAC	929	Navajas et al. 2010
	6,5KbCOIR	AATACCAGTGGGAACCGC		
ATP6-COIII	16KbATP6F	GACATATATCAGTAACAATGAG	818	
	16kBCOIIIR	GACTCCAAGTAATAGTAAAACC		
Cyt B	10KbCytbF-1	GCAGCTTTAGTGGATTTACCTAC	985	
	10KbCytbPRIM	CTACAGGACACGATCCCAAG		
COII-ATP6	6,5KBCOII	GATTATTAGTTAGATCAGCAGACG	775	
	6,5KbATP6	GTGTAAATACATAAGGTAATAACCC		

Set 1: Ten microliters of the PCR product were supplemented with 10 μl 2× loading dye and denaturized at 95 °C for 5 min. The samples were separated on 8 % polyacrylamide gels in the DCode^TM^ Universal Mutation Detection System (Bio-Rad) in the following conditions: 20 W, 340 V, and 58 mA for 3 h. The products contained in the gel were visualized using the Silver Stain Kit (Kucharczyk; Warsaw, Poland).

Set 2: The PCR matrices (20 μl) were purified with ExoSap-Exonulease I and Shrimp Alkaline Phosphatase Kits. The PCR products were directly sequenced using a BigDye Terminator Cycle Sequencing Mix v3.1 in an ABI3730xl automated DNA sequencer (Life Technologies; Warsaw, Poland).

### Global DNA methylation levels

The global DNA methylation analyses were performed using an Imprint Methylated DNA Quantification Kit MDQ1 (Sigma, USA) based on the ELISA principle. We used the 96-well plate format. DNA concentration was diluted to 150 ng/μl in the binding solution. DNA binding was achieved by incubating 30 μl diluted DNA at 37 °C for 1 h. One hundred fifty microliters of block solution were added, and the samples were incubated at 37 °C for 30 min. Next, the DNA and block solutions were removed from all the wells which were washed three times with 150 μl of 1× wash buffers. Fifty microliters of diluted capture antibody were placed in each well and incubated at room temperature for 1 h. After removing the capture antibody and washing four times with the wash buffer, each well was filled with 50 μl of diluted detection antibody. The plates were incubated at room temperature for 30 min. The detection antibody was removed from the wells which were washed five times with the wash buffer. Each well was then filled with 100 μl of developing solution and incubated at room temperature for about 10 min for color change, and subsequently 50 μl of stop solution were added. The absorbance of each sample was measured five times at 450 nm. To calculate the percentages of methylated DNA cytosine relative to the methylated control DNA, in which 100 % of cytosines are methylated, the following equation was used: [(A450S − A450B) / (A450MC − A450B)] × 100. Methylated DNA weights (in nanograms) were also computed with the following equation: [(A450S − A450B) − intercept] / slope; where A450S is the average absorbance of the sample; A450MC is the average absorbance of the methylated control DNA; A450B is the average absorbance of the blank, and the intercept and slope are equal to 0.08208 and 2.68e-3, respectively.

### Statistical analysis

The differences in DNA concentrations, percentages of DNA 5-methylcytosine, and the methylated DNA weights between the control, tau-fluvalinate-sensitive, and tau-fluvalinate-resistant *Varroa* mites were analyzed using one-way ANOVA and Tukey’s test (SAS Institute Version 9.13., 2002–2003 license 86636). Bliss transformation (*y* = arc sin (*x*/100)^0.5^) was used for percentages of DNA 5-methylocytosine. The significance of differences in the mutation incidence between the groups was estimated using the *χ*^2^ test.

## Results

**Fig. 2:**
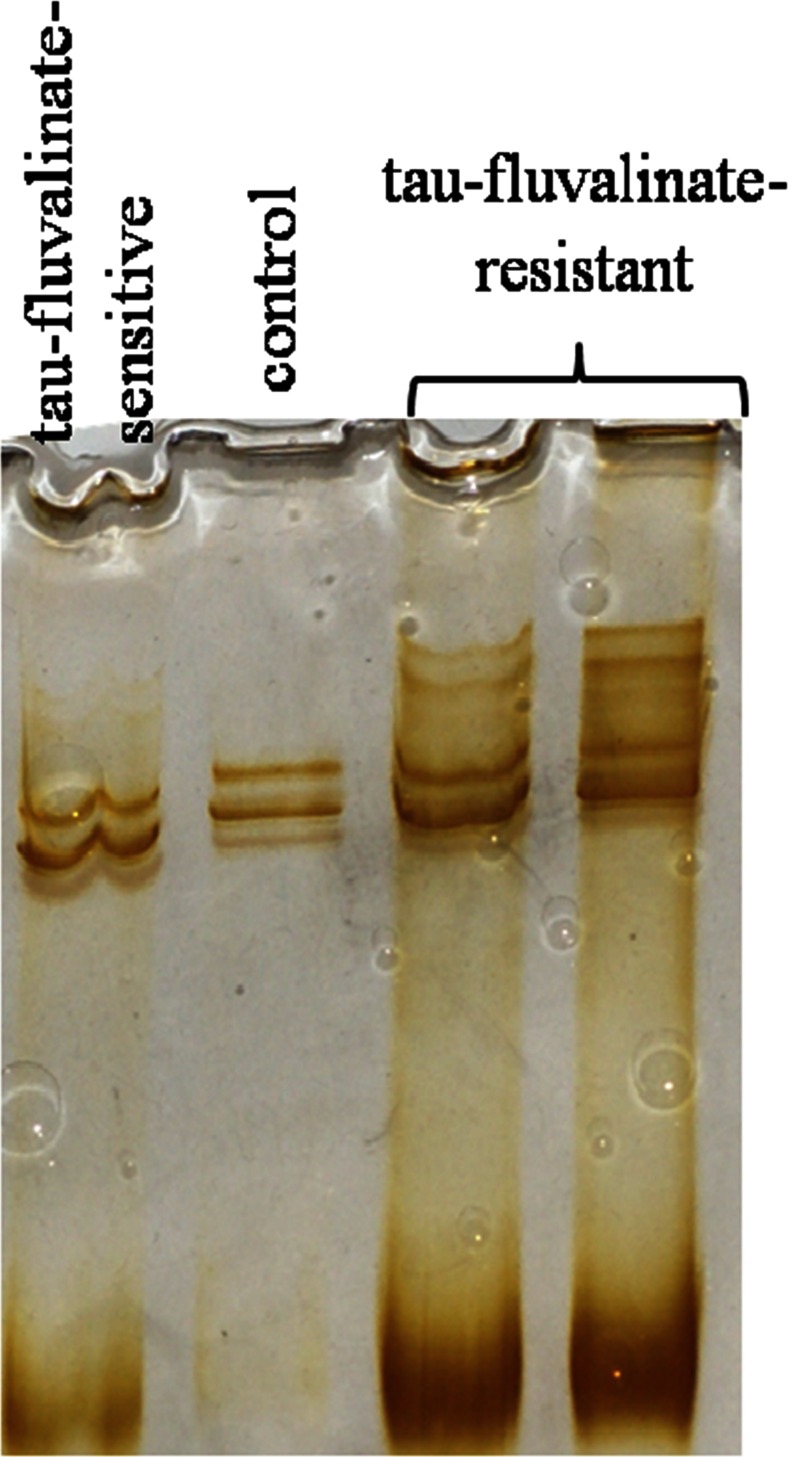
An example of a PCR-SSCP electrophorogram of the *Varroa* mites from the control, tau-fluvalinate-sensitive, and tau-fluvalinate-resistant groups

Forty tau-fluvalinate-sensitive and 35 tau-fluvalinate-resistant mites were found among the 75 *Varroa* females from the first part. Bands were observed only in the case of COI 320 bp in the PCR-SSCP electrophorograms. However, all tau-fluvalinate-resistant mites had four bands, whereas both the tau-fluvalinate-sensitive and control ones had only two bands (see Fig. 2). No differences were observed between the DNA nucleotide sequence in the control and tau-fluvalinate-sensitive groups. Sense-changing mutations were identified at the frequency of 8 % (test *χ*^2^ 12.00, *P* < 0.002) in the tau-fluvalinate-resistant mites in comparison with the two remaining groups (0 %). The sequencing results were consistent with the results of PCR-SSCP, since higher mutation frequencies corresponded with the presence of two additional bands in all the tau-fluvalinate-resistant mites (Fig. 2) as compared with the control and tau-fluvalinate-sensitive ones.

Both the DNA 5-methylcytosine percentage and the methylated DNA weight were lower in the tau-fluvalinate-resistant mites than in the control and tau-fluvalinate-sensitive ones (Table 2). Consequently, decreased DNA 5-methylcytosine percentages and methylated DNA weights corresponded with higher band numbers on the PCR-SSCP electrophorograms in the tau-fluvalinate-resistant mites.

**Table 2:** DNA concentrations, percentages of DNA 5-methylcytosine, and the methylated DNA weights in the *Varroa* mites from the control, tau-fluvalinate-sensitive, and tau-fluvalinate-resistant groups

*Varroa* mites	DNA concentration (ng/μl)	DNA methylation (%)	Weights of methylated DNA (ng)
	$\overline{x}$ ± SD	$\overline{x}$ ± SE	$\overline{x}$ ± SD
Control	5 ± 1.9	35.97^a^ ± 0.12	3.05^a^ ± 0.03
Tau-fluvalinate-sensitive	5 ± 0.0	36.17^a^ ± 0.15	3.01^a^ ± 0.01
Tau-fluvalinate-resistant	7 ± 1.0	25.01^b^ ± 0.18	2.80^b^ ± 0.02

$\overline{x}$ mean value, *SD* standard deviation, *SE* standard error, *lowercase letters* the differences are statistically significant for comparison within the columns at *P* ≤ 0.05

## Discussion

The development of resistance to pyrethroids, and other acaricides, usually appears between the fourth and seventh year of treatment in the *Varroa* mite populations (Eischen 1995; Elzen et al. 2001). Therefore, we should develop adequate methods in order to detect both the resistant mites and determine their percentages. One such method has proved to be the PCR-SSCP, which helps quickly identify resistant *V. destructor* mites by detecting mutations (compare; Hayashi 1992). This method helped us detect two additional bands in our study (Fig. 2) in the SSCP-PCR electrophorograms of the drug-resistant mites. The PCR-SSCP method seems promising, considering the fact that Sheen et al. (2009) and Cheng et al. (2007) obtained similar results for *Mycobacterium tuberculosis*, resistant to pyrazinamide, isoniazid, or rifampin.

The increased mutation rate does not explain the whole plasticity of *Varroa* organisms in response to toxic environment pressure. There are epigenetic modifications, which allow the genome to effectively respond to environmental signals; both harmful and useful (Kenyon 2005; Castonguay and Angers 2012). Tau-fluvalinate-resistant mites had lower percentages of global DNA methylation and lower methylated DNA weights (Table 2). The reduced levels of global DNA methylation in tau-fluvalinate-resistant parasites suggest activation of certain, previously inactive, genes.

Xia et al. (2012) suggested that unmethylated CpGs have a higher mutation rate in comparison with methylated CpGs. Flores et al. (2013) informed that mutations in targeting pathway of DNA methylation can reduce global genomic DNA methylation. This could be further investigated. Reduced levels of global DNA methylation may not only increase pyrethroid resistance by activating certain genes but also by increasing the mutation rate.

The DNA methylation pattern can be heritable (Holliday and Grigg 1993; Ledón-Rettig et al. 2012), and therefore, the epigenetic resistance to pyrethroids can be also heritable. Such factors as pyrethroids de novo alter the programming of the DNA methylation pattern during mite development (Flores et al. 2013). Therefore, we suggest a previously undescribed fourth mechanism (see “Introduction”) of developing pyrethroid resistance in *Varroa* mites.

The decrease in DNA methylation levels was observed in honeybees administrated such bio-stimulators as caffeine, curcumin, and coenzyme Q10 (Strachecka et al. 2014a, b, 2015a). The opposite effect was observed in the case of harmful amphotericin B (Strachecka et al. 2012) and bromofenvinphos treatments (Strachecka et al. 2015b). We observed quite the opposite effect in the *Varroa* mites. The harmful tau-fluvalinate treatment decreased DNA methylation levels. This implies that harmful chemotherapeutics can induce contradictory changes in DNA methylation mechanisms of honeybees and *Varroa* mites.

## Conclusions

The PCR-SSCP method applied to gene fragments of mitochondrial COI can be used to determine the tau-fluvalinate resistance of *V. destructor* mites. We suggest that there is a previously undescribed mechanism of developing pyrethroid resistance in *Varroa* mites. The present article is the first report about it. This paper may set new directions for studies aimed at defining drug resistance with molecular methods.
